# Continuous Glucose Monitoring Provides Durable Glycemic Benefit in Adolescents and Young Adults with Type 1 Diabetes: 12-Month Follow-Up Results

**DOI:** 10.1155/2023/6718115

**Published:** 2023-10-26

**Authors:** Kellee M. Miller, Colleen Bauza, Lauren G. Kanapka, Mark A. Clements, Daniel J. DeSalvo, Korey Hood, Laurel H. Messer, Jennifer Sherr, Katherine Bergamo, Amy Criego, Emily Freiner, Sarah K. Lyons, Roshanak Monzavi, Wayne Moore, Priya Prahalad, Jill H. Simmons, Mark Sulik, R. Paul Wadwa, Ruth S. Weinstock, Steven M. Willi, Kristen Williams, Lori M. Laffel, for the CITY Study Group

**Affiliations:** ^1^T1D Exchange, St. Petersburg, FL, USA; ^2^Jaeb Center for Health Research, Tampa, FL, USA; ^3^Children's Mercy Hospital, Kansas City, MO, USA; ^4^Baylor College of Medicine, Houston, TX, USA; ^5^Stanford University, Stanford, CA, USA; ^6^Barbara Davis Center for Diabetes, University of Colorado Anschutz Medical Campus, Aurora, CO, USA; ^7^Yale School of Medicine, New Haven, CT, USA; ^8^University of North Carolina Diabetes Care Center, Chapel Hill, NC, USA; ^9^Health Partners Institute, International Diabetes Center, St. Louis Park, MN, USA; ^10^Joslin Diabetes Center, Harvard Medical School, Boston, MA, USA; ^11^Children's Hospital Los Angeles, Los Angeles, CA, USA; ^12^Vanderbilt University Medical Center, Nashville, TN, USA; ^13^Rocky Mountain Diabetes & Osteoporosis Center, Idaho Falls, ID, USA; ^14^SUNY Upstate Medical University, Syracuse, NY, USA; ^15^Children's Hospital of Philadelphia, Philadelphia, PA, USA; ^16^Naomi Berrie Diabetes Center, Columbia University, New York City, NY, USA

## Abstract

**Objective:**

To further evaluate glycemic outcomes during the observational extension phase of the Continuous Glucose Monitoring (CGM) Intervention for Teens and Young Adults randomized clinical trial (RCT). *Subjects and Methods*. Following a 26-week RCT comparing CGM with blood glucose monitoring (BGM) in 153 adolescents and young adults aged 14 to <25 years old with suboptimally controlled type 1 diabetes, 70 (89%) participants in the BGM group initiated use of CGM (referred to as BGM–CGM cohort), and 70 (95%) participants in the CGM group continued to use of CGM (CGM–CGM cohort) for an additional 26 weeks.

**Results:**

In the CGM–CGM cohort, mean hemoglobin A1c (HbA1c) decreased from 8.9% ± 0.9% (74 ± 9.8 mmol/mol) at randomization to 8.3% ± 1.3% (67 ± 14.2 mmol/mol) at 52 weeks (*p* < 0.001); however, significant improvement in time in target range (TIR) 70–180 mg/dL was not observed from prerandomization (38% ± 13%) to 52 weeks (41% ± 18%). Median percent time <70 mg/dL decreased from 3.0% before randomization to 1.1% at 52 weeks (*p* < 0.001). In the BGM–CGM cohort, mean HbA1c decreased from 8.9% ± 1.2% (74 ± 13.1 mmol/mol) before CGM initiation to 8.5% ± 1.3% (69 ± 14.2 mmol/mol) after 26 weeks of CGM use (*p* < 0.001) and mean TIR increased from 34% ± 12% to 38% ± 15% (*p*=0.01). The median percent time <70 mg/dL decreased from 3.3% before CGM initiation to 1.2% after 26 weeks of CGM use (*p* < 0.001). No participants discontinued CGM use during the extension phase.

**Conclusions:**

This further evaluation of CGM supports the findings of the preceding RCT that use of CGM improves glycemic control and reduces hypoglycemia in adolescents and young adults with type 1 diabetes. This trial is registered with NCT03263494.

## 1. Introduction

Substantial improvements in continuous glucose monitoring (CGM) technology have demonstrated enhanced performance with both greater accuracy and convenience. These advances led to FDA approval in 2016 for individuals to use glucose values obtained by CGM for insulin dose decisions without confirmatory blood glucose monitoring (BGM) via use of a blood glucose meter [1]. The CGM Intervention for Teens and Young Adults (CITY) study was conducted to assess whether a more recent CGM model was effective in improving glycemic control among adolescents and young adults with type 1 diabetes. The 6-month randomized clinical trial (RCT) included 153 adolescents and young adults aged 14 to <25 years with type 1 diabetes at 14 US-based endocrinology centers. Findings from the RCT phase, which have been previously published, demonstrated significant reductions in hemoglobin A1c (HbA1c) (primary outcome for RCT, improvement in time in target glucose range (TIR) 70–180 mg/dL, and reductions in CGM-measured hypoglycemia among participants using real-time CGM compared with a group using standard BGM [2].

The RCT was followed by a 6-month extension phase to assess longer-term outcomes in the CGM group and to allow the BGM group to crossover to using CGM. The aim of the extension phase for the CGM cohort was to assess if the beneficial glycemic results observed during the RCT were lasting over a longer period. The aim of the extension phase for the BGM cohort was to assess if a similar glycemic benefit could be achieved following crossover from using BGM to CGM. Glycemic outcomes for both cohorts over the full 52-week period including the RCT and extension are reported herein.

## 2. Methods

The protocol and informed consent and assent forms were approved by a central Institutional Review Board (IRB) and site IRB where required. The protocol is available at https://public.jaeb.org/datasets/diabetes and details are provided on clinicaltrials.gov (NCT03263494). We followed the methods of Miller et al. [3].

The randomized trial included 153 participants, at 14 US-based centers, with a clinical diagnosis of type 1 diabetes, age 14 to <25 years, diabetes duration ≥1 year, use of either an insulin pump or multiple daily insulin injections, total daily insulin requirement ≥0.4 units/kg/day, no use of real-time CGM in the 3 months before enrollment, and HbA1c 7.5% to <11.0% (58 to <97 mmol/mol). At the start of the RCT, individuals were assigned in a random manner to either utilize CGM with Dexcom G5 or to use BGM alone. Out of the 74 participants in the CGM group, 71 successfully completed the 26-week RCT, while 79 participants were in the BGM group, with 71 of them successfully completing the trial.

A 26-week extension phase followed the 26-week RCT and included 140 of the 142 participants completing the RCT. In the extended phase of the trial, participants from the original BGM group began using CGM, forming what is referred to as the BGM–CGM cohort. Meanwhile, participants from the original CGM group continued their CGM usage, constituting the CGM–CGM cohort. During the observational extension phase, all participants used the Dexcom G6 CGM (Dexcom, Inc., San Diego, CA) instead of the Dexcom G5 due to ease of burden on participants, given the removal of need for twice daily calibrations, and availability of devices.

Throughout the 52-week follow-up period, every participant underwent in-clinic study visits at 4, 6, 13, 26, 39, and 52 weeks. In the BGM group, a masked CGM device was worn for a span of 1 week after the 13-week visit and for a period of 2 weeks leading up to the 26-week visit (with a clinic visit scheduled at the 24-week mark for the placement of the CGM device). Roughly 2 weeks after initiating CGM usage, participants attended an additional in-clinic study visit for supplementary training. Training on real-time CGM was provided using standardized materials developed for the study. In addition, CGM participants received a handout at each study visit highlighting the benefits and features of CGM, such as the reduced need for finger stick BGM and the utility of the smartphone CGM application.

Adverse events specified in the protocol as reportable were hyperglycemia involving treatment at a healthcare facility or met the Diabetes Control and Complications Trial definition for diabetic ketoacidosis [4], severe hypoglycemia (specified as an event involving altered consciousness that necessitated assistance of treatment from another person), device-related events impacting safety, and all serious adverse events irrespective of causation.

## 3. Statistical Analysis

HbA1c outcomes included continuous HbA1c and percentages of participants meeting HbA1c target (<7.0% (<53 mmol/mol) and <7.5% (<58 mmol/mol)). CGM-measured outcomes included percent of TIR (70–180 mg/dL), percent of time in hypoglycemia (<70 and <54 mg/dL), and percent of time in hyperglycemia (>180, >250, and >300 mg/dL). Mean glucose and coefficient of variation also were calculated. Furthermore, consensus guideline targets for time in various ranges were explored as a secondary outcome, including the percentage of participants with more than 70% TIR 70–180 mg/dL, the percentage of participants with less than 4% time <70 mg/dL, and less than 1% time <54 mg/dL. Frequency of hypoglycemic episodes per week, defined as 15 consecutive minutes with a sensor glucose value <54 mg/dL, was calculated. Additional analyses of glycemic outcomes included stratification by daytime (6 am to 12 midnight) and nighttime (12 midnight to 6 am) for percent TIR 70–180 mg/dL and percent of time in hypoglycemia (<70 and <54 mg/dL).

Baseline CGM-measured results were determined based on the masked data gathered over a 2-week screening phase. For subsequent visits during the use of real-time CGM, outcomes were computed by combining data from the preceding 4 weeks at the 6, 13, 26, 39, and 52-week intervals for the CGM–CGM group, and at the 26 and 39-week visits for the BGM–CGM group. Regarding the BGM cohort, CGM outcomes were calculated for the 13 and 26-week assessments using information from the 2-week duration of masked CGM usage. Central laboratory HbA1c was measured at randomization and 13, 26, 39, and 52 weeks at the University of Minnesota using the Tosoh A1c 2.2 Plus Glycohemoglobin Analyzer.

Change in glycemic outcomes within cohorts were evaluated using a paired *t*-test, signed rank test, or McNemar's test, as appropriate. Baseline measurements were assessed before randomization and analyses included only those completing the 52-week visit. Analyses were conducted with SAS software version 9.4 (SAS Institute Inc., Cary, NC) and adjustment for multiple comparisons were made using the adaptive Benjamini and Hochberg [5] procedure.

## 4. Results

Of the 153 participants who initiated the trial, 142 participants completed the 26-week RCT Of the 142 completing the RCT, 140 initiated the extension phase, 70 of 71 (99%) in the CGM–CGM cohort and 70 of 71 (99%) in the BGM–CGM cohort. All 140 participants who initiated the extension phase completed 52 weeks of follow-up. The age range of the 140 participants at the start of the extension phase was 14–25 years, 49% were female, 63% non-Hispanic white, and 54% used an insulin pump. Table S1 describes the participant characteristics for each cohort.

### 4.1. Glycemic Outcomes in CGM–CGM Cohort

**Figure 1:**
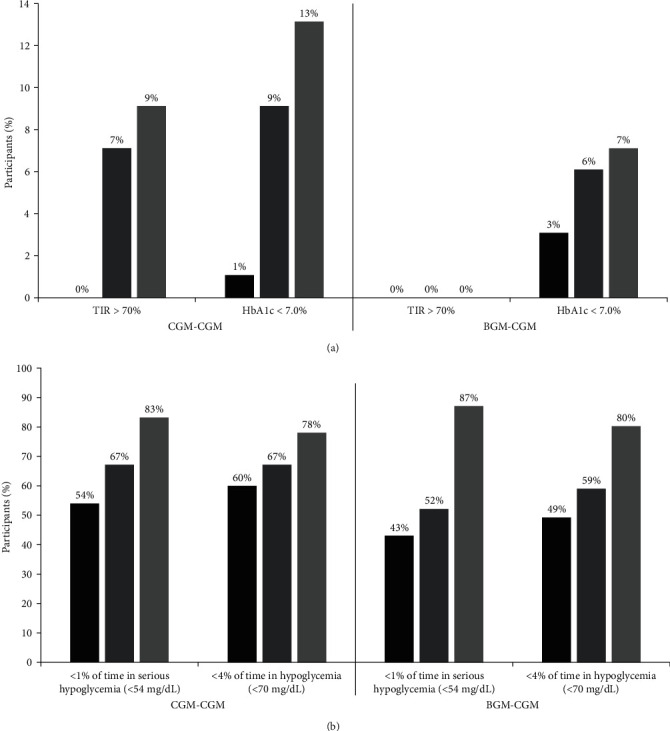
Proportion of participants achieving glycemic targets in each cohort. (a) Time-in-Range more than 70% of 24 hr period and HbA1c below 7.0%. (b) Less than 1% of time in serious hypoglycemia (<54 mg/dL) and less than 4% of time in hypoglycemia (<70 mg/dL). Solid black bar represents Baseline. Solid dark grey bar represents 26-week visit. Solid light grey represents 52-week visit.

Among the 70 individuals belonging to the CGM–CGM cohort, the average HbA1c level decreased from 8.9% ± 0.9% (74 ± 9.8 mmol/mol) during the RCT's initial baseline measurement to 8.5% ± 1.2% (69 ± 13.1 mmol/mol) at the 26-week mark, and further to 8.3% ± 1.3% (67 ± 14.2 mmol/mol) at the 52-week stage (*p* < 0.001 for RCT baseline vs. 52 weeks; *p*=0.27 for 26 vs. 52 weeks; as shown in Table 1). In addition, a notable rise was evident in the proportion of CGM–CGM participants with HbA1c levels below 7.0% (<53 mmol/mol) and below 7.5% (<58 mmol/mol) from the RCT baseline to the 52-week period (as detailed in Table 1 and shown in Figure 1(a)). The average TIR between 70 and 180 mg/dL was 38% (equivalent to 9.2 hr per day) at the start of the study, increased to 42% (equivalent to 10.2 hr per day) at the 26-week mark, and remained at 41% (equivalent to 9.8 hr per day) by the end of the 52-week period for the CGM–CGM group (*p*-values > 0.05 for baseline vs. 52 weeks and 26 vs. 52 weeks, as indicated in Table 1). Initially, before the introduction of real-time CGM, none of the participants in the CGM–CGM cohort achieved a TIR exceeding 70%, but this improved to 9% achieving more than 70% TIR by the 52-week milestone (*p*=0.03, as shown in Table 1 and Figure 1(a)). No significant alterations in TIR were observed for either daytime (6 am to 12 midnight) or nighttime (12 midnight to 6 am) hours from the beginning to the end of the 52-week period, nor from the 26-week to the 52-week stage (as detailed in Table 1). The median percentage of time spent below 70 mg/dL decreased from 3.0% (equivalent to 43 min per day) at RCT baseline to 2.1% (equivalent to 30 min per day) at the 26-week mark, and further to 1.1% (equivalent to 16 min per day) by the end of the 52-week period (*p* < 0.001 for baseline vs. 26 weeks; *p*=0.04 for 26 vs. 52 weeks; as detailed in Table 1 and Figure S1). Significant decreases also were noted in the percentage of time spent below 54 mg/dL and in the occurrence of hypoglycemic events defined by CGM data. A greater number of participants reached the hypoglycemia targets (i.e., spending less than 4% of time below 70 mg/dL and less than 1% of time below 54 mg/dL) from the study's start to the 52-week time point (as shown in Table 1 and Figure 1(a)). The reduction in time spent below 70 mg/dL and below 54 mg/dL from baseline to 52 weeks was observed when analyzed separately for daytime and nighttime hours (as detailed in Table 1). Significant changes in CGM-measured hyperglycemia (% >180, >250, and >300 mg/dL) from RCT baseline to 52 weeks were not observed in the CGM–CGM cohort.

### 4.2. Glycemic Outcomes in BGM–CGM Cohort

Out of the 70 participants within the BGM–CGM cohort, the average HbA1c level decreased from 8.9% ± 1.2% (74 ± 13.1 mmol/mol) at the initiation of the extension phase (before real-time CGM introduction) to 8.5% ± 1.3% (69 ± 14.2 mmol/mol) at the 52-week visit, which was 26 weeks after the commencement of real-time CGM usage (*p* < 0.001, as indicated in Table 2). A greater percentage of participants achieved an HbA1c level below 7.5% (<58 mmol/mol) at the conclusion of the extension phase (52 weeks) in comparison to the beginning of the extension phase (26 weeks) (as shown in Table 2). However, no notable differences were observed in the proportions of participants achieving HbA1c levels below 7.0% (<53 mmol/mol) (as detailed in Table 2 and shown in Figure 1(b)). The average percentage of time in range (TIR) between 70 and 180 mg/dL increased from 34% (equivalent to 8.1 hr per day) at the 26-week time point, before the commencement of real-time CGM, to 38% (equivalent to 9.1 hr per day) at the 52-week time point (*p*=0.01, as detailed in Table 2). None of the participants in the BGM–CGM cohort achieved a TIR exceeding 70% either at 26 weeks before real-time CGM use or at 52 weeks following 6 months of CGM use (as shown in Table 2 and Figure 1(b)). Similar improvements in TIR were observed when analyzed separately for daytime and nighttime hours (Table 2).

The median percentage of time spent below 70 mg/dL decreased from 3.3% (equivalent to 47 min per day) before CGM utilization (at 26 weeks) to 1.2% (equivalent to 18 min per day) after approximately 26 weeks of CGM usage (at 52 weeks) (*p* < 0.001 for 26 vs. 52 weeks, as shown in Table 2 and Figure S2). Significant reductions also were evident in the percentage of time spent below 54 mg/dL and in CGM-defined hypoglycemic events. Before CGM implementation at the 26-week masked CGM assessment, 52% of the BGM–CGM cohort maintained less than 1% of time below 54 mg/dL, while this proportion increased to 87% at the 52-week assessment (*p* < 0.001, as detailed in Table 2 and Figure 1(b)). A similarly noteworthy outcome was observed for the goal of less than 4% of time spent below 70 mg/dL, with 80% of participants achieving this target at 52 weeks compared with 49% at 26 weeks before CGM initiation (as indicated in Table 2 and Figure 1(b)). Reductions were observed for both CGM-measured daytime and nighttime hypoglycemia (Table 2).

### 4.3. Sensor Use and Insulin Dose for Both Cohorts

During the 28 days leading up to the 26-week assessment, participants in the CGM–CGM group utilized the Dexcom G5 CGM for a median of 79% of the time (Table 3). This proportion increased slightly to a median of 86% of the time by the 52-week mark, during which participants transitioned to using the Dexcom G6 CGM. However, this difference did not achieve statistical significance (*p*=0.17). By the 52-week point, a substantial majority of CGM–CGM participants, specifically 91% of those with 52-week visit, were still using CGM. Participants in the BGM–CGM group utilized the Dexcom G6 CGM for a median of 91% of the time during the 28 days leading up to the 52-week evaluation (as indicated in Table 3). By the 52-week time point, an overwhelming majority of BGM–CGM participants, specifically 99% of those with a 52-week visit, were still using CGM. At the 52-week mark, 88% of participants in the CGM–CGM cohort and 68% of participants in the BGM–CGM cohort were utilizing the Dexcom Mobile® application on their mobile phones to monitor their glucose levels, either alongside or instead of using the CGM receiver. In both cohorts, more than 60% of the participants opted to share their glucose readings with another individual using the Dexcom share/follow applications. As the study concluded, a majority of participants had adopted CGM for insulin dosing without relying on BGM finger stick confirmation, with 97% of individuals in both the CGM–CGM cohort and the BGM–CGM cohort making this choice. There was little to no change in total daily insulin dose per kilogram of body weight from baseline to 52 weeks in the CGM–CGM cohort (0.82–0.89 median units/kg per day) and from 26 to 52 weeks in the BGM–CGM cohort (0.89–0.84 median units/kg per day) (Table S2).

### 4.4. Safety Outcomes

Over the course of the 52-week period in the CGM–CGM cohort, there were four participants who reported severe hypoglycemic events with impaired cognition requiring assistance from another person for treatment. Among these cases, three occurred during the RCT phase, while one occurred during the extension phase. In the BGM–CGM cohort, there was one participant who reported a severe hypoglycemic event during the RCT phase, and no such events were reported during the extension phase after CGM initiation.

Within the CGM–CGM group, three participants experienced episodes of DKA during the RCT phase, and one participant experienced DKA during the extension phase. In the BGM–CGM cohort, one participant experienced DKA during the RCT phase, and there were no instances of DKA during the extension phase. A comprehensive overview of adverse events can be found in Table S3.

## 5. Discussion

This observational extension followed the completion of an RCT comparing CGM and BGM in adolescents and young adults, ages 14–25 years with type 1 diabetes. The results of the extension phase, which further evaluated CGM use, support the findings of the preceding RCT in which CGM was shown to be effective in reducing HbA1c as well as hypoglycemia; however, very few participants achieved the HbA1c glycemic target of HbA1c <7.0% (<53 mmol/mol), highlighting the challenges of self-care behaviors in adolescents and young adults with type 1 diabetes.

The extension phase included the two cohorts from the 6-month RCT, those initially randomized to CGM and to BGM. First, it is notable that >90% of both cohorts provided data for the 52-week outcomes, especially given the recognized challenges and frequent gaps in care during adolescence and young adulthood [6]. In the CGM–CGM cohort that used CGM during both the RCT and extension phase, improvements in HbA1c were maintained from 26 to 52 weeks of follow-up, without increase in hypoglycemia as the reduction in time <70 mg/dL was further reduced from 26 to 52 weeks. The cohort using BGM during the 26-week RCT phase experienced similar glycemic benefit and reduction in hypoglycemia following 26 weeks of CGM use after real-time CGM initiation at the end of the antecedent RCT. Furthermore, for this cohort the initiation and ongoing support for CGM use mirrored implementation generally delivered during routine clinical care with fewer contacts than in the first 6 months of the RCT for the CGM–CGM cohort. The results of this extension study are supported by findings from previous trials and real-world data. The DIAMOND study which evaluated CGM among 158 adults using multiple daily injections reported improvements in HbA1c and decreased time spent in hypoglycemia, however, this study used an older version of Dexcom CGM and did not include individuals <25 years of age [7]. A 7-year retrospective study examining the benefit of CGM initiation within 1 year of diagnosis among 396 individuals (94%, <18 years of age) found the observed reduction in HbA1c following CGM use was maintained over a 7-year period compared with individuals not using CGM [8, 9].

In this study, 70% of participants in both cohorts used CG Man average of 6 or more days per week at the 52-week time point. The major prior large randomized trial in this age group was the JDRF CGM RCT conducted over a decade ago [10]. In that trial, a glycemic benefit of CGM was not seen in adolescents and young adults. However, only 30% of that study's cohort used the CGM device in that study regularly (6–7 days/week), substantially lower than was seen in the current trial. In the more recent United Kingdom-based MILLENNIALS Study, 31 participants wore Dexcom G6 CGM an average of 86% of the time and significant improvements in time in range and HbA1c were observed over the 8-week crossover trial [11]. Although smaller, the MILLENNIALS study had similar CGM use and glycemic improvements to our study.

The acceptance of CGM as well as the durability of CGM use in vulnerable adolescents and young adults with type 1 diabetes speaks to the improved performance of available CGM devices that seem to offer the opportunity for improved self-care without increasing management burden [12–14].

The Dexcom mobile app was used by over two-thirds of participants at 52 weeks (CGM–CGM: 88%; BGM–CGM: 68%). The handy access to glucose levels on a smartphone also likely matches the preferences of adolescents and young adults, who prefer to appear like their peers rather than use a medical device in social settings [15]. Furthermore, at 52 weeks, nearly two-thirds of both cohorts using the mobile app were using the SHARE function that allows glucose values to be viewed by others designated by the CGM user. The ability to remotely share real-time glucose data has important implications for college-aged youth who may live away from home but still benefit from a caregiver receiving out of range alerts for low or high glucose values, as long as these follow-up reminders from the caregivers are helpful and not considered intrusive [16]. In a retrospective analysis of 15,000 youth and adolescents using Dexcom CGM, the Dexcom SHARE feature was associated with higher TIR [17]. Future research can address interventions designed to provide guidance for insulin dosing based on the CGM data as well as ways for caregivers and others receiving remote CGM data to support their loved ones with diabetes self-management.

The strengths of this study include its multicenter design (14 sites), high rate of retention, and diverse population with the study cohort including more than a third of participants from under-represented racial and ethnic groups and 42% having public insurance. However, there are a few limitations. A different Dexcom CGM system was used during the RCT (Dexcom G5) than used during the extension phase (Dexcom G6). The G6 system, unlike the G5, did not require twice daily BGM calibrations and included an automated sensor insertion device, although both the G5 and G6 allowed for nonadjunctive use. The switch to G6 may have impacted use of the device in a positive direction. Yet, the integration of the latest available technology for the observational extension phase allows the results generated to be more easily translated to clinical practice. In addition, this study did not assess other models of real-time CGM or intermittent scanning CGM.

This extension study further demonstrates that real-time CGM improves HbA1c and reduces hypoglycemia among adolescents and young adults with type 1 diabetes. The results of the 6-month RCT and the sustained benefit over the 12-month period provide strong evidence for CGM use as a standard of care for adolescents and young adults with type 1 diabetes. Continued research, innovation in therapy, and quality improvement interventions are needed to achieve clinical targets safely and effectively, including HbA1c <7.0% (<53 mmol/mol), for this population.

## Figures and Tables

**Table 1:** Glycemic outcomes in the CGM–CGM cohort.

	Baseline	26 weeks	52 weeks	*p*-Value baseline vs. 52 weeks ^*∗*^	*p*-Value 26 vs. 52 weeks ^*∗*^
HbA1c					
*N*^†^	70	70	70		
HbA1c*—mean ± SD %* (*mmol/mol*)	8.9 ± 0.9	8.5 ± 1.2	8.3 ± 1.3	<.001	0.27
	(74 ± 9.8)	(69 ± 13.1)	(67 ± 14.2)		
Change from baseline*—mean ± SD*	–	−0.4 ± 1.0	−0.5 ± 1.2	NA	NA
HbA1c < 7.0% (<53 mmol/mol)—*N* (*%*)	1 (1%)	6 (9%)	9 (13%)	0.01	0.41
HbA1c < 7.5% (<58 mmol/mol)—*N* (*%*)	3 (4%)	13 (19%)	18 (26%)	<.001	0.27
CGM-measured outcomes					
* N* ^†^	63	60	64		
Hours of data—*median* (*IQR*)	302 (269, 325)	569 (404, 639)	580 (430, 638)		
Glucose control*—mean ± SD*					
% Time in range 70–180 mg/dL	38% ± 13%	42% ± 16%	41% ± 18%	0.26	0.78
% Time in range 70–140 mg/dL^‡^	22% ± 10%	25% ± 13%	25% ± 14%	0.26	0.76
Mean glucose (mg/dL)	207 ± 36	202 ± 41	207 ± 44	0.80	0.55
Coefficient of variation (%)	41% ± 7%	38% ± 6%	37% ± 7%	<0.001	0.49
Hypoglycemia—*median* (*IQR*)					
% Time < 54 mg/dL	0.9% (0.4%, 2.9%)	0.5% (0.1%, 1.3%)	0.1% (0.0%, 0.6%)	<0.001	<0.001
% Time < 70 mg/dL	3.0% (1.2%, 7.7%)	2.1% (0.8%, 4.7%)	1.1% (0.4%, 3.1%)	<0.001	0.04
Rate of hypoglycemic events per week^§^	1.3 (0.6, 3.3)	1.0 (0.1, 2.5)	0.3 (0.0, 0.9)	<0.001	<0.001
Hyperglycemia					
% Time > 180 mg/dL*—mean ± SD*	57% ± 16%	54% ± 18%	57% ± 20%	0.80	0.49
% Time > 250 mg/dL—*median* (*IQR*)	29% (18%, 44%)	27% (13%, 36%)	29% (14%, 40%)	0.84	0.46
% Time > 300 mg/dL—*median* (*IQR*)	13% (8.0%, 26%)	11% (4.7%, 19%)	13% (4.7%, 21%)	0.74	0.54
CGM glycemic targets-*N* (*%*)					
% Time in range 70–180 mg/dL >70%^‡^	0 (0%)	4 (7%)	6 (9%)	0.03	0.41
% Time < 54 mg/dL <1%^‡^	34 (54%)	40 (67%)	53 (83%)	<0.001	0.04
% Time < 70 mg/dL <4%^‡^	38 (60%)	40 (67%)	50 (78%)	0.01	0.13
Daytime (6 am to <12 am)					
*N* ^†^	63	60	64		
Hours of data—*median* (*IQR*)	221 (199,243)	429 (306, 479)	437 (325, 475)		
Glucose control*—mean ± SD*					
% Time in range	37% ± 13%	42% ± 17%	40% ± 19%	0.23	0.83
Hypoglycemia—*median* (*IQR*)					
% Time < 54 mg/dL	0.7% (0.1%, 2.4%)	0.4% (0.0%,1.0%)	0.1% (0.0%, 0.4%)	<0.001	0.003
% Time < 70 mg/dL	2.5% (0.9%, 5.2%)	1.4% (0.5%,4.4%)	0.8% (0.2%, 2.5%)	<0.001	0.08
Nighttime (12 am to <6 am)					
*N* ^†^	63	60	64		
Hours of data—*median* (*IQR*)	78 (70, 84)	140 (98,159)	147 (108,161)		
Glucose control*—mean ± SD*					
% Time in range	41% ± 16%	45% ± 18%	43% ± 19%	0.44	0.87
Hypoglycemia—*median* (*IQR*)					
% Time < 54 mg/dL	1.2% (0.0%, 5.9%)	0.6% (0.0%, 2.2%)	0.2% (0.0%, 0.9%)	<0.001	0.04
% Time < 70 mg/dL	3.6% (0.8%, 12%)	2.5% (0.4%, 6.0%)	1.5% (0.2%, 4.0%)	<0.001	0.27

*Note*.  ^*∗*^*p*-Value from a paired *t*-test, signed rank test, or McNemar's test, as appropriate. Only includes participants who had values at both time points. *p*-Values are adjusted for multiple comparisons to control the false discovery rate. ^†^*N* includes participants completing 52-week visit with available data.^‡^Outcomes percent time in range (70–140 mg/dL), percentages of participants >70% percent time in range (70–180 mg/dL), <4% percent time <70 mg/dL, and <1% percent time <54 mg/dL were added post hoc. ^§^A CGM-measured hypoglycemic event was defined as 15 consecutive minutes with a sensor glucose value <54 mg/dL. The end of the hypoglycemic event was defined as a minimum of 15 consecutive minutes with a sensor glucose concentration >70 mg/dL [18].

**Table 2:** Glycemic outcomes in the BGM–CGM cohort.

	RCT baseline	26 weeks (before CGM initiation)	52 weeks (26-weeks CGM use)	*p*-Value 26 vs. 52 weeks ^*∗*^
HbA1c				
*N*^†^	69	69	69	
HbA1c—*mean ± SD %* (*mmol/mol*)	8.8 ± 0.9	8.9 ± 1.2	8.5 ± 1.3	<0.001
	(73 ± 9.8)	(74 ± 13.1)	(69 ± 14.2)	
Change from baseline—*mean ± SD*	NA	NA	−0.4 ± 0.8	NA
HbA1c < 7.0% (<53 mmol/mol)—*N* (*%*)	2 (3%)	4 (6%)	5 (7%)	0.56
HbA1c < 7.5% (<58 mmol/mol)—*N* (*%*)	6 (9%)	7 (10%)	14 (20%)	0.04
CGM-measured outcomes				
*N*^†^	69	69	69	
Hours of data—*median* (*IQR*)	313 (279, 376)	292 (253, 315)	609 (429, 641)	
Glucose control—*mean ± SD*				
% Time in range 70–180 mg/dL	37% ± 11%	34% ± 12%	38% ± 15%	0.01
% Time in range 70–140 mg/dL^‡^	22% ± 9%	20% ± 9%	22% ± 11%	0.02
Mean glucose (mg/dL)	210 ± 35	220 ± 35	217 ± 45	0.51
Coefficient of variation (%)	43% ± 7%	42% ± 8%	38% ± 6%	<0.001
Hypoglycemia—*median* (*IQR*)				
% Time < 54 mg/dL	1.4% (0.3%, 3.1%)	0.9% (0.3%, 2.3%)	0.2% (0.0%, 0.6%)	<0.001
% Time < 70 mg/dL	4.2% (1.8%, 7.3%)	3.3% (1.1%, 6.4%)	1.2% (0.5%, 3.2%)	<0.001
Rate of hypoglycemic events per week^§^	1.7 (0.7, 3.5)	1.6 (0.5, 2.8)	0.3 (0.0, 0.9)	<0.001
Hyperglycemia				
% Time > 180 mg/dL—*mean ± SD*	58% ± 15%	62% ± 14%	60% ± 16%	0.18
% Time > 250 mg/dL—*median* (*IQR*)	32% (23%, 43%)	36% (25%, 46%)	29% (21%, 44%)	0.18
% Time > 300 mg/dL—*median* (*IQR*)	17% (11%, 28%)	21% (13%, 28%)	14% (7.5%, 28%)	0.37
CGM glycemic targets-*N* (*%*)				
% Time in range 70–180 mg/dL >70%^‡^	0 (0%)	0 (0%)	0 (0%)	NA
% Time <54 mg/dL <1%^‡^	30 (43%)	36 (52%)	60 (87%)	<0.001
% Time <70 mg/dL <4%^‡^	34 (49%)	41 (59%)	55 (80%)	<0.001
Daytime (6 am to <12 am)				
*N* ^†^	69	69	69	
Hours of data—*Median* (*IQR*)	231 (203, 278)	215 (189, 237)	455 (333, 481)	
Glucose control*—Mean ± SD*				
% Time in range	36% ± 12%	33% ± 12%	37% ± 15%	0.01
Hypoglycemia—*median* (*IQR*)				
% Time < 54 mg/dL	1.2% (0.3%, 3.3%)	0.9% (0.3%, 1.8%)	0.1% (0.0%, 0.3%)	<0.001
% Time < 70 mg/dL	2.8% (1.5%, 7.0%)	2.4% (1.2%, 5.8%)	1.0% (0.4%, 2.4%)	<0.001
Nighttime (12 am to <6 am)				
*N* ^†^	69	69	69	
Hours of data—*median* (*IQR*)	83 (74, 101)	76 (65, 81)	152 (101,161)	
Glucose control—*mean ± SD*				
% Time in range	39% ± 15%	35% ± 16%	41% ± 18%	0.01
Hypoglycemia—*median* (*IQR*)				
% Time < 54 mg/dL	1.4% (0.1%, 3.8%)	0.6% (0.0%, 4.3%)	0.1% (0.0%, 0.7%)	<0.001
% Time < 70 mg/dL	4.9% (1.8%, 7.5%)	3.1% (0.6%, 6.8%)	1.4% (0.3%, 3.5%)	<0.001

*Note*.  ^*∗*^*p*-Value from a paired *t*-test, signed rank test, or McNemar's test, as appropriate. Only includes participants who had values at both time points. *p*-Values are adjusted for multiple comparisons to control the false discovery rate.^†^*N* includes participants completing 52-week visit with available data. ^‡^Outcomes percent time in range (70–140 mg/dL), percentages of participants >70% percent time in range (70–180 mg/dL), <4% percent time <70 mg/dL, and <1% percent time <54 mg/dL were added post hoc.^§^A CGM-measured hypoglycemic event was defined as 15 consecutive minutes with a sensor glucose value <54 mg/dL. The end of the hypoglycemic event was defined as a minimum of 15 consecutive minutes with a sensor glucose concentration >70 mg/dL [18].

**Table 3:** CGM use and device feature use.

	CGM–CGM						BGM–CGM	
		RCT phase		Extension phase		*p*-Value 26 vs. 52 weeks^a^	Extension phase	
CGM use	6 weeks	13 weeks	26 weeks	39 weeks	52 weeks		39 weeks	52 weeks
Avg # days CGM use per week—median (Q1, Q3)	7.0 (6.1, 7.0)	6.8 (4.8, 7.0)	6.8 (4.9, 7.0)	6.8 (5.8, 7.0)	6.9 (5.3, 7.0)	0.12	7.0 (6.0, 7.0)	7.0 (5.8, 7.0)
CGM use <5 days	10 (16%)	16 (25%)	16 (25%)	9 (14%)	14 (22%)		9 (13%)	15 (22%)
CGM use ≥5 days	54 (84%)	48 (75%)	48 (75%)	55 (86%)	50 (78%)		60 (87%)	54 (78%)
CGM use—% of time								
*N*^b^	70	70	70	70	70		70	70
* Median* (*Q1*, *Q3*)	86% (64%, 94%)	76% (41%, 92%)	79% (46%, 94%)	88% (64%, 95%)	86% (55%, 95%)	0.17	90% (75%, 96%)	91% (62%, 95%)
0%	2 (3%)	3 (4%)	6 (9%)	5 (7%)	6 (9%)		2 (3%)	1 (1%)
<50%	7 (10%)	16 (23%)	13 (19%)	6 (9%)	10 (14%)		6 (9%)	10 (14%)
50% to <60%	8 (11%)	4 (6%)	5 (7%)	2 (3%)	3 (4%)		1 (1%)	6 (9%)
60% to <70%	2 (3%)	6 (9%)	6 (9%)	6 (9%)	6 (9%)		4 (6%)	4 (6%)
70% to <80%	8 (11%)	8 (11%)	6 (9%)	8 (11%)	3 (4%)		9 (13%)	4 (6%)
80% to <90%	12 (17%)	12 (17%)	11 (16%)	10 (14%)	14 (20%)		13 (19%)	9 (13%)
90% to ≤100%	31 (44%)	21 (30%)	23 (33%)	33 (47%)	28 (40%)		35 (50%)	36 (51%)
Device feature use								
*N*^c^	67	66	64	65	64		68	69
Mobile application	–	49 (74%)	52 (81%)	–	56 (88%)	–	–	47 (68%)
SHARE feature among mobile application users	–	28 (50%)	32 (62%)	–	35 (63%)	–	–	34 (72%)
Dosing insulin using CGM without BG confirmation	61 (91%)	58 (88%)	63 (98%)	63 (97%)	62 (97%)	–	66 (97%)	67 (97%)

*Note*. ^a^*p*-Values are from a signed-rank test. *p*-Values are adjusted for multiple comparisons to control the false discovery rate (FDR). ^b^Only participants who completed and had data available at the 52-week visit are included in the tabulation of CGM use. ^c^Only participants who had nonzero and nonmissing CGM use are included in the tabulation of feature use.

## Data Availability

Data supporting the results can be found at https://public.jaeb.org/datasets/diabetes.

## Supplementary Materials

Supplementary Materials Figure S1: time in hypoglycemia (average minutes/day <70 mg/dL). Figure S2: time in hypoglycemia (average minutes/day <54 mg/dL). Table S1: participant characteristics at the start of the extension phase (26 weeks). Table S2: insulin use in CGM–CGM and BGM–CGM cohorts. Table S3: adverse events by cohort and phase.
