# Supplementary material for: Continuous Glucose Monitoring Provides Durable Glycemic Benefit in Adolescents and Young Adults with Type 1 Diabetes: 12-Month Follow-Up Results
Source: Pediatr Diabetes. 2023 Oct 26;2023:6718115. doi: 10.1155/2023/6718115 (PMC12016685; doi:10.1155/2023/6718115)
Supplement: Supplementary Materials — Figure S1: time in hypoglycemia (average minutes/day <70 mg/dL). Figure S2: time in hypoglycemia (average minutes/day <54 mg/dL). Table S1: participant characteristics at the start of the extension phase (26 weeks). Table S2: insulin use in CGM–CGM and BGM–CGM cohorts. Table S3: adverse events by cohort and phase. [file 6718115.f1.docx]

# Online Supplemental Material

**Supplemental Figure 1. Time in Hypoglycemia (Average Minutes/day <70 mg/dL)**


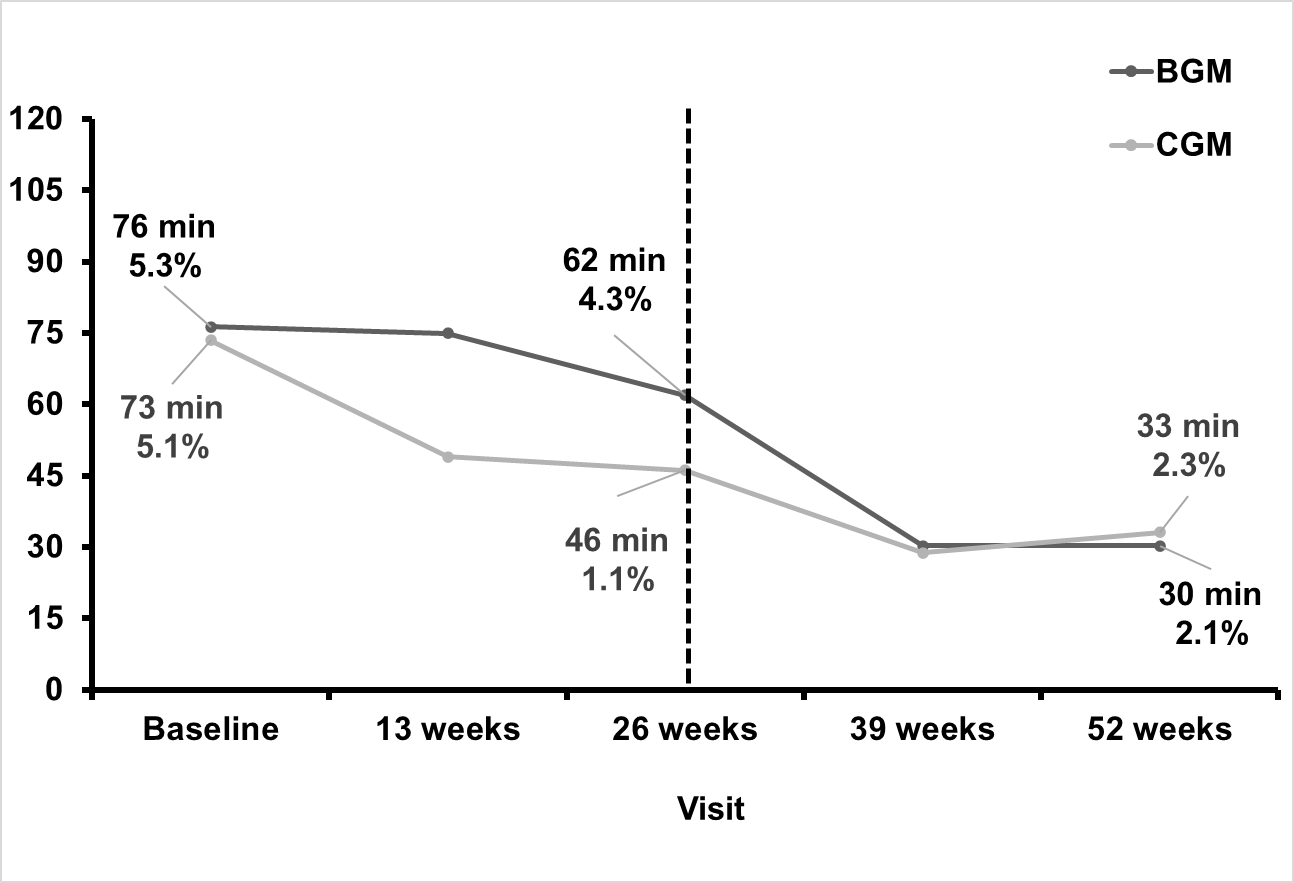


Legend: Dots represent average minutes per day spent <70 mg/dL. Solid black represents BGM group. Solid grey represents CGM group.

**Supplemental Figure 2. Time in Hypoglycemia (Average Minutes/day <54 mg/dL)**

Legend: Dots represent average minutes per day spent <70 mg/dL. Solid black represents BGM group. Solid grey represents CGM group.

# Supplemental Table 1. Participant Characteristics at Start of Extension Phase (26 weeks)

|  | **Overall^1^**  **(N=140)** | **CGM-CGM^a^**  **(N=70)** | **BGM-CGM^a^**  **(N=70)** |
| --- | --- | --- | --- |
| **Age (years)** |  |  |  |
| 14-<19 | 88 (63%) | 45 (64%) | 43 (61%) |
| 19-<25 | 52 (37%) | 25 (36%) | 27 (39%) |
| *Median (Q1, Q3)* | 17.8 (16.1, 20.6) | 17.8 (15.8, 20.5) | 17.9 (16.4, 20.7) |
| *Range* | 14.6 to 25.5 | 14.6 to 25.5 | 14.6 to 25.5 |
| **Diabetes Duration (years)** |  |  |  |
| *Median (Q1, Q3)* | 8.5 (5.8, 13.9) | 8.2 (5.7, 13.2) | 9.7 (6.1, 14.3) |
| *Range* | 1.7 to 21.7 | 1.7 to 21.3 | 1.9 to 21.7 |
| **Gender: Female^c^** – *N (%)* | 68 (49%) | 30 (43%) | 38 (54%) |
| **Race/ethnicity^c^** – *N (%)* |  |  |  |
| White non-Hispanic | 87 (63%) | 46 (67%) | 41 (59%) |
| Black non-Hispanic | 9 (6%) | 2 (3%) | 7 (10%) |
| Hispanic or Latino | 31 (22%) | 17 (25%) | 14 (20%) |
| Asian | 6 (4%) | 1 (1%) | 5 (7%) |
| American Indian/Alaskan Native | 1 (<1%) | 0 (0%) | 1 (1%) |
| More than one race | 5 (4%) | 3 (4%) | 2 (3%) |
| **Annual Household Income^c^** – *N (%)* |  |  |  |
| < $25,000 | 10 (9%) | 6 (11%) | 4 (7%) |
| $25,000 - <$35,000 | 20 (18%) | 7 (13%) | 13 (22%) |
| $35,000 - <$50,000 | 17 (15%) | 4 (7%) | 13 (22%) |
| $50,000 - <$75,000 | 18 (16%) | 8 (15%) | 10 (17%) |
| $75,000 - <$100,000 | 16 (14%) | 11 (20%) | 5 (9%) |
| $100,000 - <$200,000 | 27 (24%) | 15 (27%) | 12 (21%) |
| ≥ $200,000 | 5 (4%) | 4 (7%) | 1 (2%) |
| **Health Insurance^c^** – *N (%)* |  |  |  |
| Private | 81 (58%) | 41 (59%) | 40 (57%) |
| Public | 58 (42%) | 28 (41%) | 30 (43%) |
| **Insulin Route** – N (%) |  |  |  |
| Injections | 64 (46%) | 36 (51%) | 28 (40%) |
| Pump | 76 (54%) | 34 (49%) | 42 (60%) |
| **HbA1c^d^ (%)** |  |  |  |
| *Mean ± SD % (mmol/mol)* | 8.7 ± 1.2 (72±13.1) | 8.5 ± 1.2 (69±13.1) | 8.9 ± 1.2 (69±13.1) |
| *Range* | 6.1 to 12.6 | 6.1 to 12.0 | - 1. to 12.6 |

^a^Only 140 participants who completed extension for the RCT Treatment groups are included in this table.

^b^Missing data: Race/ethnicity 1 (<1%), annual household income 27 (19%), health insurance 1 (<1%)

^c^Only assessed at screening.

^d^26 week central lab value where available, otherwise the local screening value.

# Supplemental Table 2. Insulin Use in CGM-CGM and BGM-CGM Cohorts

|  | **CGM-CGM** | | **BGM-CGM** | |
| --- | --- | --- | --- | --- |
|  | N | Median (Q1, Q3) | N | Median (Q1, Q3) |
| **Basal Insulin Units per Kg** |  |  |  |  |
| Baseline | 64 | 0.42 (0.32, 0.48) | 62 | 0.41 (0.35, 0.50) |
| 26 Weeks | 62 | 0.40 (0.32, 0.47) | 60 | 0.41 (0.35, 0.51) |
| 52 Weeks | 63 | 0.39 (0.33, 0.49) | 60 | 0.43 (0.35, 0.51) |
| **Bolus Insulin Units per Kg** |  |  |  |  |
| Baseline | 64 | 0.40 (0.31, 0.67) | 62 | 0.41 (0.33, 0.57) |
| 26 Weeks | 62 | 0.48 (0.35, 0.68) | 60 | 0.44 (0.33, 0.58) |
| 52 Weeks | 63 | 0.49 (0.32, 0.77) | 60 | 0.42 (0.28, 0.57) |
| **Total Daily Insulin Units per Kg** |  |  |  |  |
| Baseline | 64 | 0.82 (0.66, 1.13) | 62 | 0.83 (0.73, 0.96) |
| 26 Weeks | 63 | 0.93 (0.68, 1.12) | 60 | 0.89 (0.68, 1.09) |
| 52 Weeks | 64 | 0.89 (0.71, 1.22) | 60 | 0.84 (0.67, 1.04) |
| **# Short-acting Injections per Day – MDI users** |  |  |  |  |
| Baseline | 35 | 4.0 (3.0, 4.0) | 25 | 4.0 (3.0, 5.0) |
| 26 Weeks | 35 | 4.0 (3.0, 5.0) | 25 | 4.0 (3.0, 4.0) |
| 52 Weeks | 35 | 4.0 (3.0, 5.0) | 25 | 4.0 (4.0, 5.0) |
| **# Bolus doses per Day – Pump users** |  |  |  |  |
| Baseline | 28 | 4.0 (3.0, 5.0) | 37 | 4.0 (3.0, 5.0) |
| 26 Weeks | 25 | 5.0 (3.0, 7.0) | 36 | 4.0 (3.0, 4.0) |
| 52 Weeks | 26 | 5.0 (4.0, 6.0) | 36 | 4.0 (3.0, 5.0) |

# Supplemental Table 3. Adverse Events by Cohort and Phase

|  | **# of events (# of participants with one or more event)** | | | |
| --- | --- | --- | --- | --- |
|  | **CGM-CGM** | | **BGM-BGM** | |
|  | **Randomized Phase**  **(N=74)** | **Extension Phase**  **(N=70)** | **Randomized Phase**  **(N=79)** | **Extension Phase**  **(N=70)** |
| **Severe hypoglycemic events^a^** | 3 (3) | 1 (1) | 1 (1) | 0 (0) |
| **Diabetic ketoacidosis events^b^** | 3 (3) | 1 (1) | 1 (1) | 0 (0) |
| **Severe hyperglycemia events^c^** | 4 (4) | 2 (2) | 5 (5) | 1. (0) |

^a^Severe hypoglycemia is defined as an event that required assistance of another person due to altered consciousness to actively administer carbohydrate, glucagon, or other resuscitative actions.

^b^Diabetic Ketoacidosis (as defined by the DCCT) involves all of the following symptoms such as polyuria, polydipsia, nausea, or vomiting; serum ketones >1.5 mmol/L or large/moderate urine ketones; either arterial blood pH <7.30 or venous pH <7.24 or serum bicarbonate <15; and treatment provided in a health care facility

^c^Severe hyperglycemia events includes any hyperglycemia or ketosis event reported that did not meet definition for DKA
